# Deciphering the sub-Golgi localization of glycosyltransferases via 3D super-resolution imaging

**DOI:** 10.1247/csf.24008

**Published:** 2024-07-11

**Authors:** Hirokazu Yagi, Seigo Tateo, Taiki Saito, Yusaku Ohta, Emiko Nishi, Saemi Obitsu, Tatsuya Suzuki, Supaphorn Seetaha, Charles Hellec, Akihiko Nakano, Takuro Tojima, Koichi Kato

**Affiliations:** 1 Graduate School of Pharmaceutical Sciences, Nagoya City University, 3-1 Tanabe-dori, Mizuho-ku, Nagoya 467-8603, Japan; 2 Exploratory Research Center on Life and Living Systems (ExCELLS), 5-1 Higashiyama, Myodaiji-cho, Okazaki 444-8787, Japan; 3 Institute for Molecular Science, National Institutes of Natural Sciences, 5-1 Higashiyama, Myodaiji-cho, Okazaki 444-8787, Japan; 4 Department of Biochemistry, Faculty of Science, Kasetsart University, Bangkok, 10900, Thailand; 5 Live Cell Super-Resolution Imaging Research Team, RIKEN Center for Advanced Photonics, 2-1 Hirosawa, Wako 351-0198, Japan

**Keywords:** Golgi apparatus, glycosyltransferase, 3D super-resolution imaging, N-glycosylation

## Abstract

The Golgi apparatus, a crucial organelle involved in protein processing, including glycosylation, exhibits complex sub-structures, i.e., *cis*-, medial, and *trans*-cisternae. This study investigated the distribution of glycosyltransferases within the Golgi apparatus of mammalian cells via 3D super-resolution imaging. Focusing on human glycosyltransferases involved in N-glycan modification, we found that even enzymes presumed to coexist in the same Golgi compartment exhibit nuanced variations in localization. By artificially making their N-terminal regions [composed of a cytoplasmic, transmembrane, and stem segment (CTS)] identical, it was possible to enhance the degree of their colocalization, suggesting the decisive role of this region in determining the sub-Golgi localization of enzymes. Ultimately, this study reveals the molecular codes within CTS regions as key determinants of glycosyltransferase localization, providing insights into precise control over the positioning of glycosyltransferases, and consequently, the interactions between glycosyltransferases and substrate glycoproteins as cargoes in the secretory pathway. This study advances our understanding of Golgi organization and opens avenues for programming the glycosylation of proteins for clinical applications.

## Introduction

The Golgi apparatus, a key organelle responsible for processing and dispatching protein products from the endoplasmic reticulum (ER), consists of flattened, membrane-enclosed compartments known as cisternae (Glick and Nakano, 2009; Nakano, 2022; Nakano and Luini, 2010; Pantazopoulou and Glick, 2019). These cisternae form stacked structures in most eukaryotic cells, exhibiting variations in size, number, morphology, and subcellular distribution based on the cell type. The Golgi stack displays directionality, with the end proximal to the ER termed the *cis* cisterna, the opposite end as the *trans* cisterna, and the intermediate region collectively referred to as the cisterna. Beyond the *trans* cisterna, there is an extension known as the *trans*-Golgi network. Proteins exiting the ER are transported as cargoes from the *cis* side to the *trans* side of the Golgi apparatus and undergo posttranslational modifications, including glycosylation, which play a pivotal role in determining their final destination and functions.

Protein glycosylation occurs stepwise and is orchestrated by a series of enzymes within the Golgi apparatus. In N-glycosylation, a major protein glycosylation process, high-mannose-type glycans attached to proteins in the ER are trimmed by mannosidases in the Golgi apparatus. Subsequently, the glycan structure undergoes diversification through the actions of enzymes responsible for the transfer of N-acetylglucosamine, galactose, fucose, sialic acid, and others. This intricate process of glycosylation involves organized enzymes; for example, mannosidases responsible for N-glycan trimming are localized on the *cis* side, whereas glycosyltransferases that diversify the outer-branch structures of glycans, are presumed to be distributed on the *trans* side, as revealed via immuno-gold electron microscopy (Rabouille *et al.*, 1995). Beyond protein glycosylation, the Golgi apparatus serves as a site for the glycosylation of lipids and elongation of glycosaminoglycan (GAG) chains in proteoglycans. In *Drosophila*, proteins involved in these distinct glycosylation processes are arranged in different Golgi stacks scattered throughout the cytoplasm (Yano *et al.*, 2005). In mammalian cells, the Golgi apparatus forms a single extensive structure called the Golgi ribbon, composed of interconnected Golgi stacks. Consequently, the overall architecture of the Golgi ribbon and the distribution of enzymes within it remain largely unknown.

Therefore, this study aimed to contribute to the technical foundation addressing this issue by employing super-resolution microscopy to precisely observe the distribution of glycosyltransferases within the Golgi apparatus of cultured mammalian cells. We focused on human glycosyltransferases involved in the diversification of the nonreducing terminal structures of N-glycans, including galactosylation [β-1,4-galactosyltransferase/B4GALT1 (GB41)], fucosylation (4-galactosyl-N-acetylglucosaminide 3-α-L-fucosyltransferase 9/FUT9), and sialylation [CMP-N-acetylneuraminate-β-1,4-galactoside α-2,3-sialyltransferase/ST3GAL3 (SA33), CMP-N-acetylneuraminate-β-galactosamide-α-2,3-sialyltransferase 4/ST3GAL4 (SA34), and β-galactoside α-2,6-sialyltransferase 1/ST6GAL1 (SA61)], using β-1,3-galactosyltransferase/B3GALT6 (GB36), a glycosyltransferase involved in GAG elongation, as a reference enzyme. All these enzymes are type II membrane proteins with an N-terminal region composed of a cytoplasmic, transmembrane, and stem segment (CTS) followed by a luminal catalytic domain. Through the quantification of the degrees of colocalization of these enzymes, we aimed to explore the factors determining their distributions in the Golgi apparatus.

## Materials and Methods

### Recombinant protein expression vectors

All plasmids used in this study are summarized in Supplementary Table 1, along with Benchling links containing the plasmid sequences and maps.

cDNA construction: The open reading frames of human B4GALT1, B3GALT6, ST3GAL3, ST3GAL4, ST6GAL1, and FUT9 were amplified via PCR using cDNA from HEK293T cells as a template. The C-terminal mNeonGreen- or mScarlet-I-fused glycosyltransferases were constructed as follows. The open reading frame sequences were replaced with Flag-AktPH and JNK Kinase Translocation Reporter (JNKKTR) in pCAGGS-Flag-AkPH-mNeonGreen and pCAGGS-JNKKTR-mScarlet-I, respectively, which were provided by Prof. Kazuhiro Aoki (ExCELLS). Here and in the figures, glycosyltransferases fused with C-terminal mNeonGreen or mScarlet-I are indicated by superscripting their names with G or S, respectively. We prepared B4GALT1^G^, B3GALT6^G^, ST3GAL3^G^, ST3GAL4^G^, ST6GAL1^G^, FUT9^G^, B4GALT1^S^, B3GALT6^S^, ST3GAL3^S^, ST3GAL4^S^, ST6GAL1^S^, and FUT9^S^. Additionally, the DNA fragments coding for chimeric glycosyltransferase mutants (designated as X-Y), composed of the N-terminal CTS region of one enzyme (X) and the C-terminal catalytic domain of another enzyme (Y), were purchased from Fasmac Co. Ltd (Atsugi, Japan), followed by insertion at the EcoRI/NotI sites of mammalian pCAGGS-Flag-AkPH-mNeonGreen and pCAGGS-JNKKTR-mScarlet-I. We prepared B3GALT6-B4GALT1^G^, B4GALT1-B3GALT6^G^, ST3GAL4-ST6GAL1^S^, and ST6GAL1-ST3GAL4^S^. Plasmids encoding iRFP-ST-CTS (CTS region of human ST6GAL1, Met1-Glu45) (SA61-CTS^R^) (Tojima *et al.*, 2024) and mScarlet-I-Rab1 (Rab1^S^) under the control of the CMV promoter were generated from mCherry-ST-CTS (Addgene, #55133), EGFP-Rab1 (Addgene, #49467), iRFP713 (Addgene, #31857), and mScarlet-I-Giantin (Giantin^S^) (Addgene, #85050) using the In-Fusion Cloning kit.

### Cell culture and transfection

Expi293F cells (Thermo Fisher Scientific, Waltham, MA, USA) were maintained in Expi293 Expression Medium (Thermo Fisher Scientific) under 8% CO_2_ at 37°C on an orbital shaker platform. For cDNA transfection, cells were plated on a 35-mm glass-bottom dish (AGC Techno Glass Co., Ltd., Yoshida, Japan) coated with poly-L-lysine (Sigma, St. Louis, MO, USA) the day before and transfected with the expression plasmids using Lipofectamine 3000 (Thermo Fisher Scientific). After overnight culture, cells expressing fluorescent protein-fused glycosyltransferases were observed using a super-resolution microscope.

### Microscopy

The cells were observed at 37°C using our previously developed super-resolution confocal live imaging microscopy (SCLIM) technique (Kurokawa and Nakano, 2020; Tojima *et al.*, 2023). The imaging system consists of an inverted microscope (IX73; Evident, Tokyo, Japan) equipped with solid-state lasers emitting at 473 nm (Blues^TM^, 50 mW; Cobolt, Stockholm, Sweden), 561 nm (Jive^TM^, 50 mW; Cobolt), and 671 nm (CL671-100-S, 100 mW; CrystaLaser, Reno, NV, USA), a 100× objective (UPlanXApo, oil, NA 1.45; Evident), a custom-built piezo actuator (Yokogawa Electric, Musashino, Japan), a high-speed spinning-disk confocal scanner (CSU-10; Yokogawa Electric), a custom-built emission splitter unit, three image intensifiers (Hamamatsu Photonics, Hamamatsu, Japan) with custom-built cooling systems, and three EM-CCD cameras (ImagEM; Hamamatsu Photonics) for green, red, and far-red channels. For 3D (XYZ) observation, sequential optical XY-slices covering the full thickness of the Golgi ribbon (approximately 6–15 μm) were collected at 0.2-μm intervals at 4–15 frames/s. Z-stack images were converted into 3D voxel data and subjected to deconvolution (fast restoration) with Volocity software (Perkin Elmer) using a theoretical point-spread function for spinning-disk confocal microscopy. The 3D images were visualized using the “3D opacity” function of Volocity.

### Image analyses

For colocalization analysis, Pearson’s correlation coefficient values were calculated using Volocity. The region of interest was set to cover the entire Golgi ribbon, and the signal threshold was determined using the Costes method (Costes *et al.*, 2004).

Multidimensional scaling (MDS) was used to create a visual map of data points based on their distances from each other. This method facilitates the creation of a Golgi atlas depicting the localization of enzymes by analyzing pairwise distances between them. The pairwise distances were estimated based on normalized Pearson’s correlation coefficient values (*r*) using the formula: *d* = 1 – *r*. These distance values are presented in Supplementary Table 2. Based on the distance table, MDS was performed using the Python module manifold from sklearn with the key parameters employed in this analysis: n_components = 3, metric = True, dissimilarity = “precomputed,” random_state = 6, and normalized_stress = “auto.” This method was based on the assumption that the coordinates of the enzymes in the atlas were represented in three-dimensional space. Subsequently, the 3D mapping was converted to 2D data using principal component analysis (PCA). The X and Y axes represent the first and second principal components of MDS 3D mapping, respectively.

We conducted image analysis using Fiji, a comprehensive distribution of ImageJ equipped with numerous plugins tailored for scientific image analysis. To segment the images, we applied the Otsu or MaxEntropy thresholding algorithm to each of the images in the green and red channels. Pixels with intensity values above the respective threshold for green or red channel images were classified as foreground, while those below were considered background. To quantify the discrepancy in distribution in the Golgi between glycosyltransferases, we employed the Jaskolski’s algorithm, Colocalization Colormap (Jaskolski *et al.*, 2005) (Supplementary Fig. 1). Specifically, we calculated the normalized mean deviation product (nMDP), which mathematically represents the correlation between intensities of corresponding pixels with values ranging from –1 to 1. The algorithm computes the index of correlation (Icorr), indicating the fraction of positively correlated (colocalized) pixels in the analyzed images, thus providing a highly sensitive quantitative measure of colocalization. In contrast, the index Inega-corr represents a fraction of negatively correlated pixels where green and red molecules are not colocalized (Inega-corr = 1 – Icorr).

## Results

### Sub-Golgi localization diversity of glycosyltransferases

**Fig. 1:**
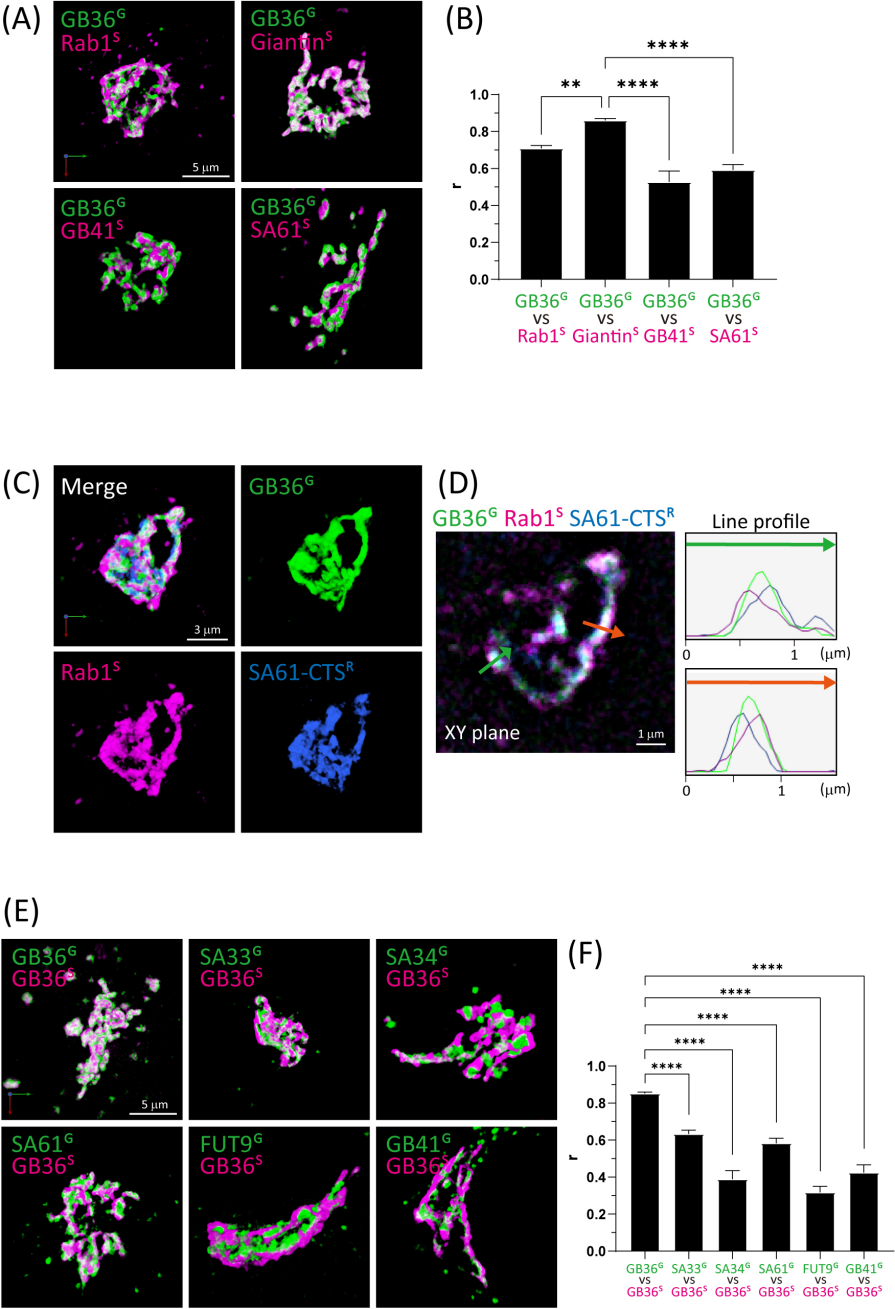
3D images of B3GALT6 in Expi293F cells (A) Dual-color 3D SCLIM images of the Golgi ribbon area of Expi293F cells expressing B3GALT6^G^ and mScarlet-I-Rab1 (Rab1^S^) (*cis*-Golgi marker), mScarlet-I-Gianitin (Giantin^S^) (medial-Golgi marker), B4GALT1^S^, or ST6GALT1^S^ (*trans*-Golgi markers). Bar, 5 μm. (B) Colocalization analysis quantified based on Pearson’s correlation coefficient values. Data are means of at least eight cells. Error bars indicate SEM. Statistical significance (p) was assessed by Dunnett’s multiple comparison test. (C, D) Triple-color (C) 3D and (D) 2D SCLIM images of the Golgi ribbon area of Expi293F cells expressing B3GALT6^G^, Rab1^S^ (*cis*-Golgi marker), and SA-CTS-iRFP (SA61-CTS^R^, *trans*-Golgi marker). Bar, 3 μm. In D, the fluorescence intensity profile across the bar for green, magenta, and blue channels in triple-color 3D imaging is shown in the graph. Bar, 1 μm. (E) Dual-color 3D SCLIM images of the Golgi ribbon area of an Expi293F cell expressing B3GALT6^S^ and B3GALT6^G^, B4GALT1^G^, ST3GAL3^G^, ST3GAL4^G^, ST6GAL1^G^, or FUT9^G^. Bar, 5 μm. (F) Colocalization analysis quantified based on Pearson’s correlation coefficient values. Data are means of at least ten cells. Error bars indicate SEM. Statistical significance (*p*) was assessed by Dunnett’s multiple comparison test, with significance levels indicated as follows: **p<0.01 and ****p<0.0001. Key: GB41, B4GALT1; GB36, B3GALT6; SA33, ST3GAL3; SA34, ST3GAL4; SA36, ST3GAL6; SA61, ST6Gal1.

We attempted to refine the estimation of 3D colocalization between two enzymes fused with distinct fluorescent proteins using dual-color SCLIM imaging. To validate our approach, we assessed the degrees of colocalization of the reference enzyme B3GALT6 (GB36) fused to mNeonGreen with the Golgi markers Rab1, Giantin, and B4GALT1 (GB41) fused to mScarlet-I. Fig. 1 illustrates that B3GALT6 predominantly colocalized with the medial-Golgi marker Giantin (Martinez-Menárguez *et al.*, 2001), whereas its degree of colocalization with the *cis*-Golgi marker Rab1 (Tisdale *et al.*, 1992) or the *trans*-Golgi marker B4GALT1 (Roth and Berger, 1982) was notably lower. Additionally, we examined the localization of B3GALT6 compared with the CTS region of human ST6GAL1 (SA61), a commonly used *trans*-Golgi marker, which revealed a significantly low degree of colocalization (Fig. 1C, D). In addition, the line profiles revealed that B3GALT6 was situated between *cis*- and *trans*-Golgi markers (Fig. 1D). These findings consistently place B3GALT6 in the medial Golgi, aligning with previously reported findings (Tie *et al.*, 2018).

Furthermore, we compared the localization of mScarlet-I-fused B3GALT6 with five glycosyltransferases [ST3GAL3 (SA33), ST3GAL4 (SA34), ST6GAL1 (SA61), FUT9, and B4GALT1 (GB41)] presumed to be more *trans*-Golgi oriented than B3GALT6 (Fig. 1E, F and Supplementary Movie 1). Colocalization with B3GALT6 was consistently low for all these enzymes. Nevertheless, the degrees of colocalization with B3GALT6 exhibited nonuniform variations, implying nuanced diversity in the sub-Golgi localization of these enzymes.

**Fig. 2:**
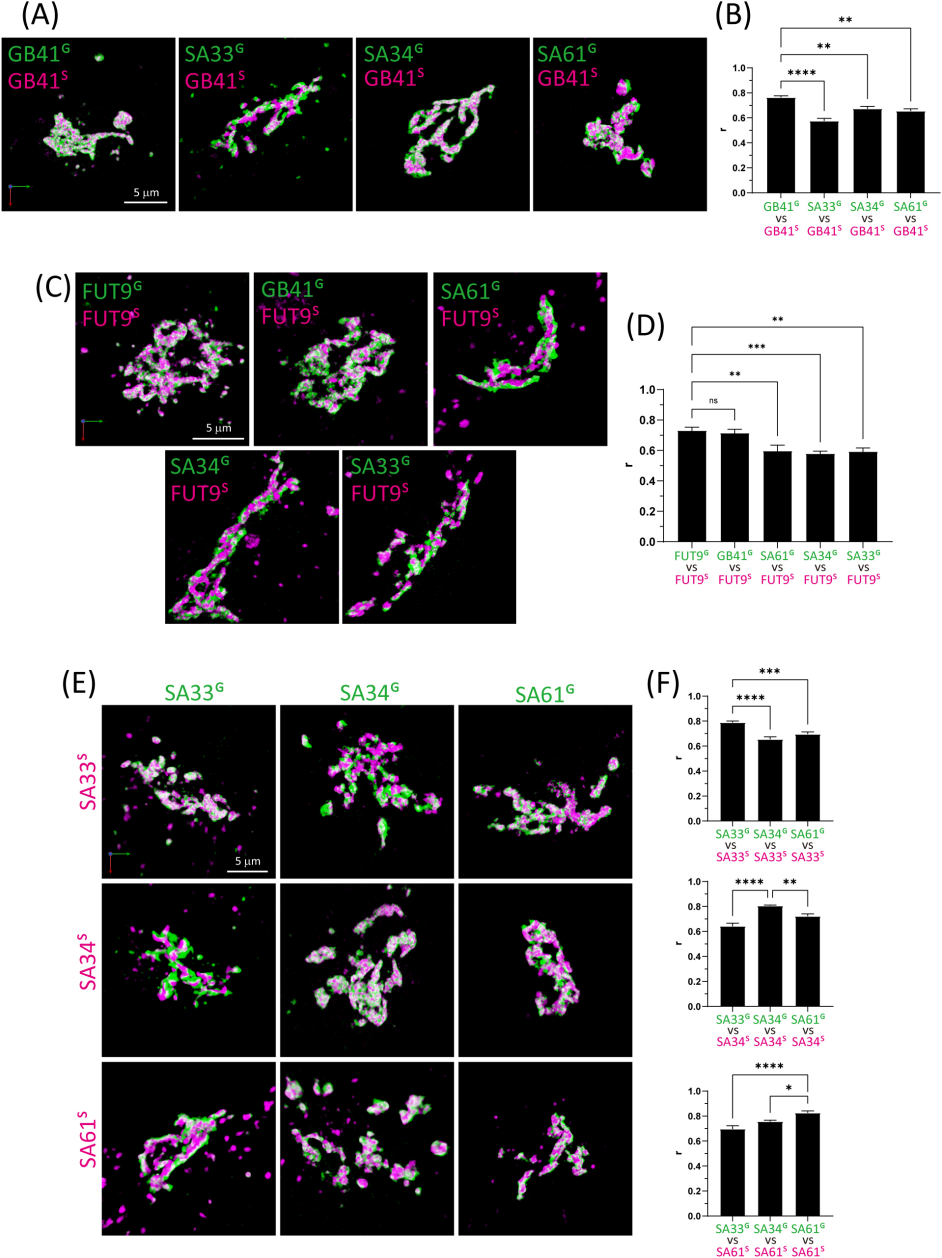
3D images of B4GALT1, ST3GAL3, ST3GAL4, ST6GAL1, and FUT9 (A, C, E) Dual-color 3D images of the Golgi ribbon area of Expi293F cells expressing two glycosyltransferases. Bars, 5 μm. (A) B4GALT1^S^ versus B4GALT1^G^, ST3GAL3^G^, ST3GAL4^G^, or ST6GAL1^G^. (C) FUT9^S^ versus FUT9^G^, B4GALT1^G^, ST3GAL3^G^, ST3GAL4^G^, or ST6GAL1^G^. (E) Upper panels: ST3GAL3^S^ versus ST3GAL3^G^, ST3GAL4^G^, or ST6GAL1^G^. Middle panels: ST3GAL4^S^ versus ST3GAL3^G^, ST3GAL4^G^, or ST6GAL1^G^. Lower panels: ST6GAL1^S^ versus ST3GAL3^G^, ST3GAL4^G^, or ST6GAL1^G^. (B, D, F) Colocalization analysis quantified based on Pearson’s correlation coefficient values. Data are means of at least ten cells. Error bars indicate SEM. Statistical significance (*p*) was assessed using Dunnett’s multiple comparison test, with significance levels indicated as follows: *p<0.05, **p<0.01, ***p<0.001, and ****p<0.0001. Key: GB41, B4GALT1; GB36, B3GALT6; SA33, ST3GAL3; SA34, ST3GAL4; SA36, ST3GAL6; SA61, ST6Gal1.

**Fig. 3:**
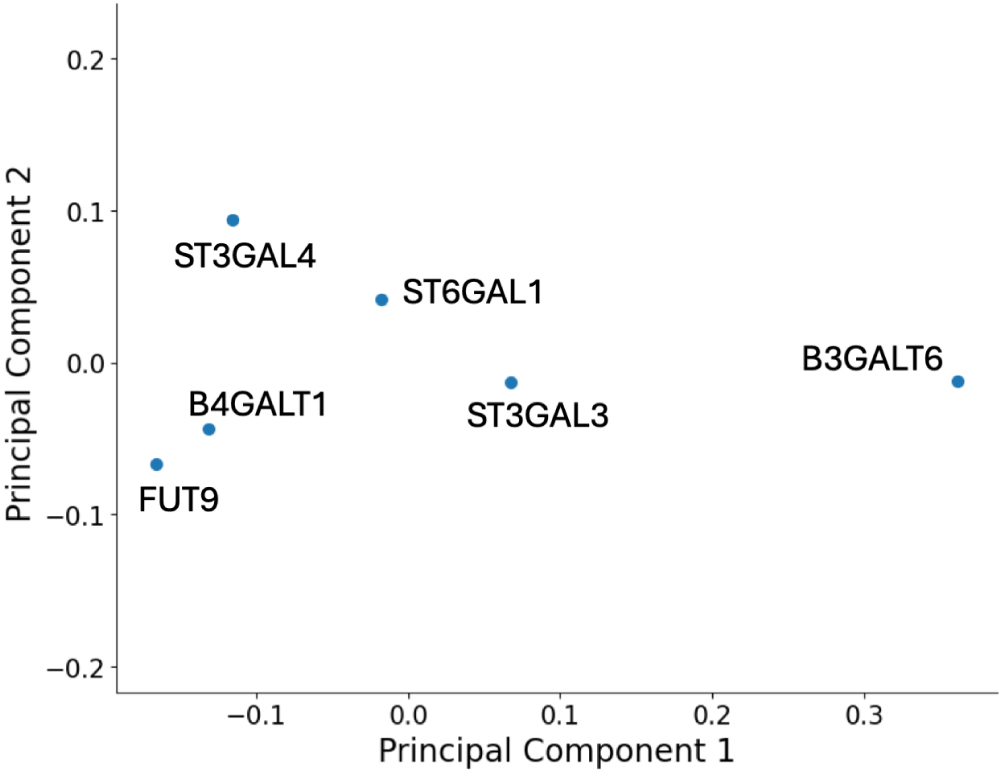
Visualization of distance relationships from the colocalization of glycosyltransferases via dimensionality reduction The mapping was generated using MDS combined with PCA (see Materials and Methods). The distance between data points reflects the differences in localization patterns of the enzymes as evaluated by imaging results (Supplementary Table 2). The X and Y axes represent the first and second principal components derived from the three-dimensional MDS plotting based on the localization patterns.

Encouraged by the feasibility of our method for sub-Golgi investigations, we extended our approach to quantify the degree of colocalizations between two glycosyltransferases selected from the *trans*-Golgi group (Fig. 2). B4GALT1 and FUT9 exhibited a higher degree of colocalization (Fig. 2C, D), contrasting with consistently low degrees of colocalization with sialyltransferases. Moreover, the degrees of colocalization among the three sialyltransferase isozymes were nonuniform, with ST3GAL3 showing a lower degree of colocalization compared to ST3GAL4 and ST6GAL1 (Fig. 2E, F and Supplementary Movie 2). Further distinctions in sub-Golgi localization were observed between ST3GAL4 and ST6GAL1. Fig. 3 provides a summary of the distance relationships among the glycosyltransferases, estimated from their colocalization degrees using MDS. Furthermore, we computed the nMDP, a very sensitive quantitative measure of colocalization, for all images (Supplementary Fig. 1A–F). The Inega-corr, the fraction of negatively correlated pixels, consistently showed higher values when comparing different glycosyltransferases than in the control group, where the same glycosyltransferase was labeled with different fluorophores. This indicates that each glycosyltransferase, even when overexpressed, exhibits a distinct distribution pattern within the Golgi.

### Determinants of glycosyltransferase localization

Considering the obtained results, we investigated the factors influencing the divergent localization of these enzymes. As previously mentioned, the CTS region of rat ST6GAL1 is known for its *trans*-Golgi localization, which mirrors that of the native enzyme. Additionally, it has been suggested that the functional localization of fucosyltransferase 6 is determined by the CTS region (Grabenhorst and Conradt, 1999). These observations prompted us to investigate whether the CTS region plays a decisive role in determining enzyme localization.

**Fig. 4:**
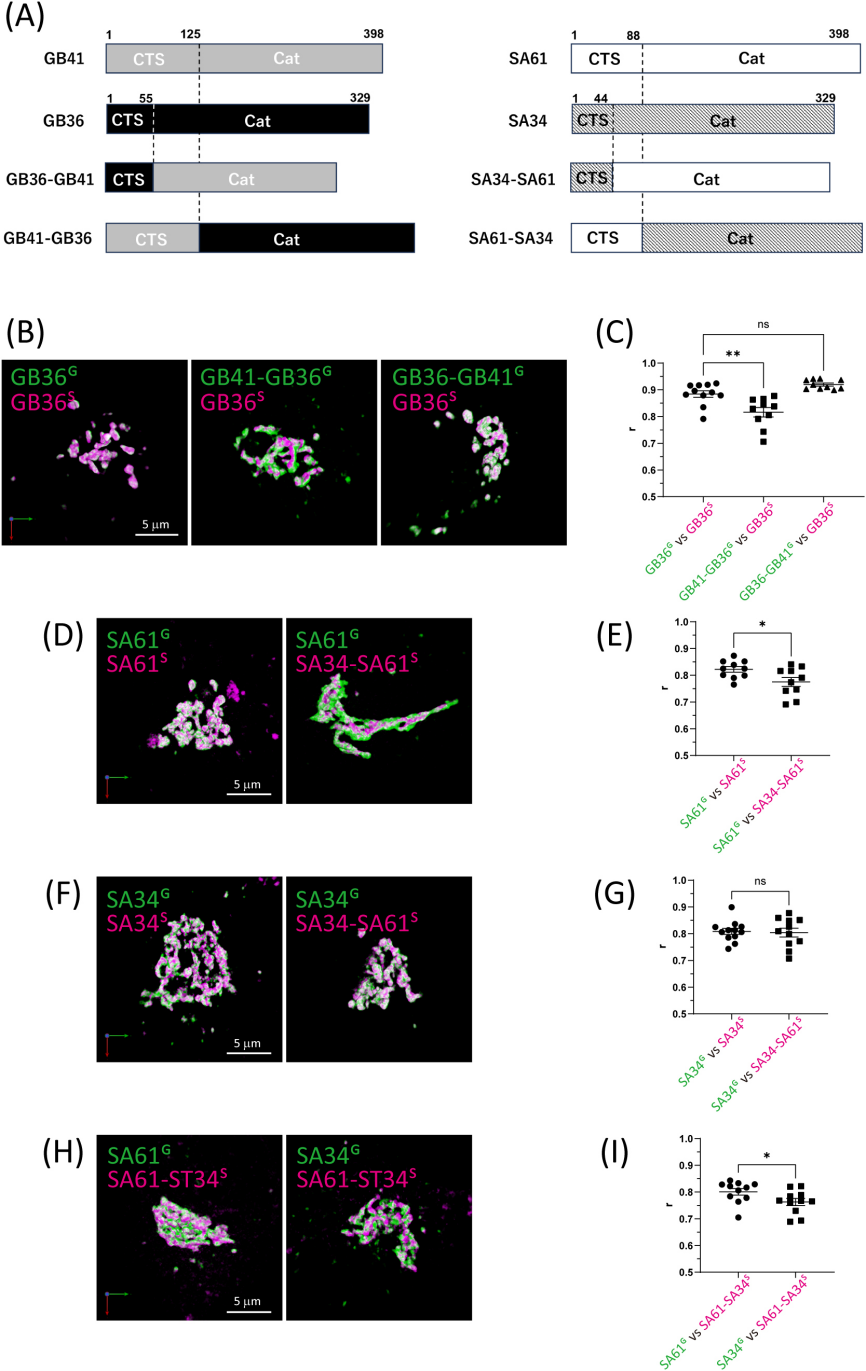
3D images of chimeric glycosyltransferase mutants (A) Schematic representation of chimeric glycosyltransferases used in this study. (B, D, F, H) Dual-color 3D images of the Golgi ribbon area of Expi293F cells expressing (B) B3GALT6^S^ versus B3GALT6^G^, B4GALT1-B3GALT6^G^, or B3GALT6-B4GALT1^G^, (D) ST6GAL1^G^ versus ST6GAL1^S^ or ST3GAL4-ST6GAL1^S^, (F) ST3GAL4^G^ versus ST3GAL4^S^ or ST3GAL4-ST6GAL1^S^, (H) ST6GAL1-ST3GAL4^R^ versus ST6GAL1^G^ or ST3GAL4^G^. Bars, 5 μm. (C, E, G, H) Colocalization analysis quantified based on Pearson’s correlation coefficient values. Data are means of at least ten cells. Error bars indicate SEM. Statistical significance (*p*) was assessed by (C) Dunnett’s multiple comparison test and (E, G, I) unpaired *t*-test with significance levels indicated as follows: *p<0.05 and **p<0.01. Key: GB41, B4GALT1; GB36, B3GALT6; SA33, ST3GAL3; SA34, ST3GAL4; SA36, ST3GAL6; SA61, ST6Gal1; GB41-GB36, B4GALT1-B3GALT6; GB36-GB41, B3GALT6-B4GALT1; SA34-SA61, ST3GAL4-ST6GAL1; SA61-SA34, ST6GAL1-ST3GAL4.

To address this inquiry, we engineered CTS-swapping enzymes between B3GALT6 and B4GALT1, the most distal enzymes in the relationship map (Fig. 3), and quantified their degree of colocalization with their respective original enzymes (Fig. 4A). The findings revealed that enzymes sharing the CTS region, rather than the catalytic domain, exhibited a higher degree of colocalization (Fig. 4B, C). Furthermore, we investigated whether the notable difference in sub-Golgi localization between ST3GAL4 and ST6GAL1 could be attributed to their CTS regions by employing CTS-swapping enzymes. Consistently, the results indicated a higher degree of colocalization when sharing the CTS region, aligning with our earlier observations (Fig. 4D–I). We also calculated the nMDP and obtained consistent results (Supplementary Fig. 1G–J). This comprehensive analysis highlights the crucial role of the CTS region in influencing enzyme localization patterns.

## Discussion

This study demonstrated that glycosyltransferases presumed to coexist within the same Golgi compartment might exhibit slight variations in their localization. Moreover, it suggests that the determining factor for these distribution differences lies in their CTS region. Notably, replacing the CTS region of the *trans*-Golgi marker B4GALT1 with that of B3GALT6 can enhance their colocalization.

Cytoplasmic tails within certain glycosyltransferases are known to directly engage with COPI coat subunits through their CTS regions, indirectly interacting with COPI adaptors such as GOLPH3/Vps74 and facilitating their retention in the Golgi apparatus (Welch and Munro, 2019). For instance, the “MRLLRR” sequence in GALNT6 directly interacts with δ-COP, leading to its localization in the *cis*-Golgi (Liu *et al.*, 2018). Intriguingly, B3GALT6, but not B4GALT1, shares a sequence with high similarity, “MKLLRR,” suggesting a potential interaction with δ-COP and localization in the medial Golgi. Additionally, it has been reported that ST3GAL4 and ST6GAL1 commonly interact with GOLPH3 through their cytoplasmic tail regions (L-X-X-R/K), whereas B4GALT1 does not participate in such interactions (Isaji *et al.*, 2014). These observations align with our findings, indicating closer localization of ST3GAL4 and ST6GAL1 (Fig. 3).

Furthermore, our analysis of CTS-swapping mutants between ST3GAL4 and ST6GAL1 revealed that these two glycosyltransferases exhibit closely but distinctively localized patterns on their CTS region (Fig. 4C, D). This suggests the existence of alternative localization mechanisms beyond interactions with COPI adaptors. It is plausible that their positioning is influenced by interactions involving the transmembrane segment and lipids, as well as interactions between the stem region and Golgi-resident proteins. Therefore, while the GOLPH3-recognizing sequence in the CTS is considered a key molecular determinant of the sub-Golgi localization of glycosyltransferases, additional regions and factors likely contribute to this intricate process.

Differences in the distribution of glycosyltransferases at the sub-Golgi level appear to be closely associated with the mechanisms of glycosylation. It is not merely a matter of enzymes involved in the early stages of N-glycan processing being located on the *cis* side and those involved in the later stages on the *trans* side. As suggested by a study on *Drosophila* cells (Yano *et al.*, 2005), or as implied by the distinct localization between B3GALT6 and B4GALT1 in the present study (Fig. 4C, D), proteins involved in glycan formation for different classes of glycoconjugates seem to exhibit distinct distributions within the Golgi apparatus. Moreover, the variations in distribution among sialyltransferase family enzymes within the *trans*-Golgi suggest the possibility that these enzymes interact with different cargoes as clients through the potential regulation of their transport routes within the Golgi apparatus. Indeed, it has been reported that different α2,3-sialylation enzymes act on different substrates in HeLa cells (Qi *et al.*, 2020).

We previously demonstrated the incorporation of molecular codes within cargoes, enhancing transport efficiency by facilitating interactions with specific cargo receptors or promoting interactions with particular glycosyltransferases along the secretory pathway (Saito *et al.*, 2022; Yagi *et al.*, 2020). Conversely, this study revealed that the molecular codes determining the sub-Golgi localization of glycosyltransferases are carried within their respective CTS regions. The revision and integration of molecular codes for cargoes and glycosyltransferases provide precise control over the positioning of glycosyltransferases, cargo transport routes, and consequently, the interactions between glycosyltransferases and cargoes in the secretory pathway. This refined approach opens avenues for programming protein glycosylation, thereby advancing the sophistication of glycoprotein expression for clinical applications.

## Funding

This work was supported in part by JST-CREST (grant number JP MJCR21E3 to T.T. and K.K.), JST FOREST Program (grant Number JPMJFR2255), the Human Glycome Atlas Project, MEXT/JSPS KAKENHI (grant numbers JP22K06213 to T.T., JP23H00382 to A.N., JP17H06414 and JP21H02625 to H.Y. and JP20K21495 and JP24H00599 to K.K.), JSPS International Fellowships for Research in Japan (PE19029 to H.C.), Grant-in-Aid for Scientific Research on Innovative Areas ― Platforms for Advanced Technologies and Research Resources “Advanced Bioimaging Support”, the ExCELLS Advanced Co-creation Platform (Spatiotemporal atlas of dynamic structure and function of organelles, 23EXC601 to H.Y.), and ExCELLS “Golgi Atlas Project” (to K.K.).

## Author Contributions

HY, AN, TT, and KK conceived and designed experiments; T Saito, EN, SO, T Suzuki, SS, and CH performed the vector construction; T Saito, ST, EN and SO and TT performed the microscope experiments; ST, YO, T Saito, and TT performed the image analysis; HY, TT, and KK wrote the paper.

## Conflict of Interest

The authors declare that they have no conflicts of interest regarding the contents of this article.

## Data Availability

The supporting information for this article is available in J-STAGE Data.

## Abbreviations

CTS: cytoplasmic, transmembrane, and stem segment
ER: endoplasmic reticulum
GAG: glycosaminoglycan
GB36: β-1,4-galactosyltransferase/B3GALT6
GB41: β-1,4-galactosyltransferase/B4GALT1
MDS: multidimensional scaling
SA33: CMP-N-acetylneuraminate-β-1,4-galactoside α-2,3-sialyltransferase/ST3GAL3
SA34: CMP-N-acetylneuraminate-β-galactosamide-α-2,3-sialyltransferase 4/ST3GAL4
SA61: β-galactoside α-2,6-sialyltransferase 1/ST6GAL1
SCLIM: super-resolution confocal live imaging microscopy

## Supplementary Material

Supplementary Movie 1 3D images of B3GALT6 and FUT9. Dual-color 3D SCLIM images of the Golgi ribbon area of an Expi293F cell expressing B3GALT6^S^ (magenta) and FUT9^G^ (green).

Supplementary Movie 2 3D images of ST3GAL3 and ST3GAL4. Dual-color 3D SCLIM images of the Golgi ribbon area of an Expi293F cell expressing ST3GAL3^S^ (magenta) and ST3GAL4^G^ (green).
