# Supplementary material for: Deciphering the sub-Golgi localization of glycosyltransferases via 3D super-resolution imaging
Source: Cell Struct Funct. 2024 Jul 11;49(2):47–55. doi: 10.1247/csf.24008 (PMC11926406; doi:10.1247/csf.24008)
Supplement: Supplementary file 3 — Supplemental Materials [file csf_49_24008_3.pdf]

Supplementary Fig. 1

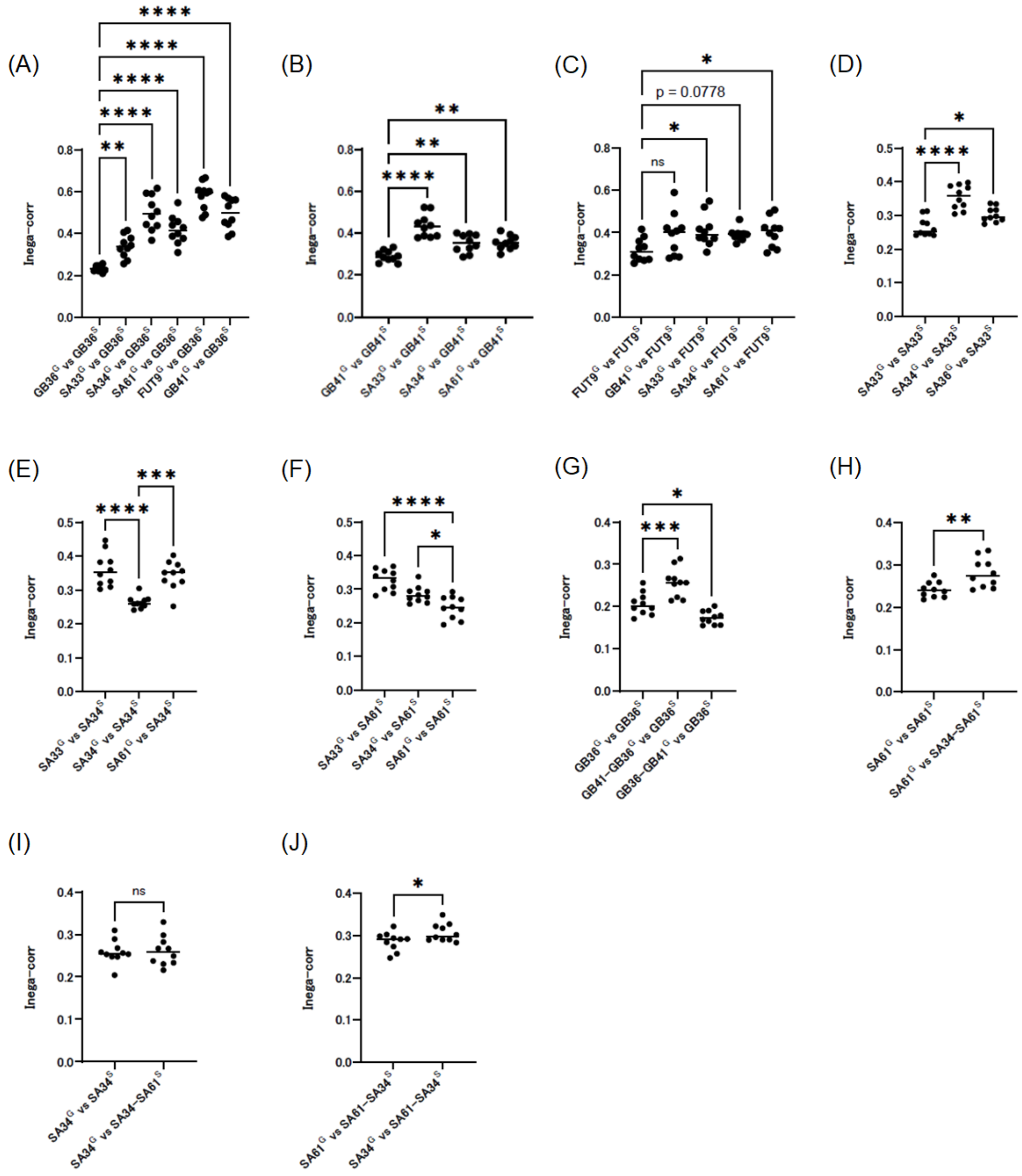

Quantification of the index of correlation (Inega-corr) as a measure of segregation of localization.

The normalized mean deviation product (nMDP) mathematically represents the correlation between the intensities of corresponding pixels, which values ranging from -1 to 1. The algorithm computes the index of correlation (Icorr), which represents the fraction of positively correlated (colocalized) pixels in the analyzed images. This allows for a very sensitive quantitative measurement of

colocalization. Conversely, the index Inega-corr represents the fraction of negatively correlated pixels, indicating where green and red molecules are not colocalized (Inega-corr = 1- Icorr). Data are presented as means of at least ten cells, with error bars indicating the standard error of the mean (SEM). Statistical significance (p) was assessed using Dunnett's multiple comparison test, with significance levels indicated as follows: * $p < 0.05$, ** $p < 0.01$, *** $p < 0.001$, and ****$p < 0.0001$.

Supplementary Table 1. List of plasmids used in this study

**Plasmid name**	**Source**	**Benchling link**
pCAGGS-Flag-AkPH-mNeonGreen	Gift from Prof. Kazuhiro Aoki (ExCELLS)	https://benchling.com/ncu/f/lib_U5UUyL86-vector/seq_xozw6KU1-pcaggs-flag-aktph-mneongreen/edit
pCAGGS-JNKKTR-mScarlet-I	Gift from Prof. Kazuhiro Aoki (ExCELLS)	https://benchling.com/ncu/f/lib_U5UUyL86-vector/seq_xYcdC0AE-pcaggs-jnkktr-mscarlet/edit
iRFP-ST-CTS	Tojima et al. eLife, 2024, 13:e92900.	
mCherry-ST-CTS	Addgene, #55133	
mScarlet-I-Rab1	This study	https://benchling.com/s/seq-GDUF2IA0A8g9D6eR0ZXl?m=slm-vhrwnHvbguX7jDcTkwqw
EGFP-Rab1	Addgene, #49467	
iRFP713	Addgene, #31857	
mScarlet-I-Giantin (Giantin^{S})	Addgene, #85050	
B4GALT1^{G}	This study	https://benchling.com/s/seq-5LFLK7AapO4DQmsYGbgk?m=slm-FGAHZ35C6gva9VNoJBfI
B3GALT6^{G}	This study	https://benchling.com/ncu/f/lib_uIJIFcXy-mneogreen-vectors/seq_W0uFajWJ-13_b3galt6-mneogreen-flanking/edit
ST3GAL3^{G}	This study	https://benchling.com/ncu/f/lib_uIJIFcXy-mneogreen-vectors/seq_MV2yWU7c-2_st3gal3-mneogreen-flanking/edit
ST3GAL4^{G}	This study	https://benchling.com/s/seq-6zfbllJj9WRvARBbgzRm?m=slm-ycgFETEfEcJBfsK18xf4

ST6GAL1^{G}	This study	https://benchling.com/s/seq-MPcCdwFzhgr6almQdvha?m=slm-TNzIDTibPSRTLuvCXloI
FUT9^{G}	This study	https://benchling.com/s/seq-OOVPVNGgpsQZasSLFlmO?m=slm-y4199L20443L9mswrmlf
B4GALT1^{S}	This study	https://benchling.com/s/seq-2Kqa1JOMJ5PFJ3wvkUfZ?m=slm-R3Xr97Gjo8qM4LzoU6Uf
B3GALT6^{S}	This study	https://benchling.com/ncu/f/lib_QwZsoSJW-suzuki/seq_WH4imTCd-pcaggs-b3galt6-mscarlet/edit
ST3GAL3^{S}	This study	https://benchling.com/ncu/f/lib_j0zhRcRt-st3gal3/seq_mV0SjZSY-pcaggs-st3gal3-mscarlet/edit
ST3GAL4^{S}	This study	https://benchling.com/s/seq-Tsc5FYLY1XuPZEYayKAp?m=slm-m4TBVmozV6gOFijAkA6k
ST6GAL1^{S}	This study	https://benchling.com/s/seq-goLKitvGldQnnFbLpU4h?m=slm-WSCL6DWi22BK7p7XZc6r
FUT9^{S}	This study	https://benchling.com/s/seq-X5kMTJLdY6SfmD8dJOBb?m=slm-sGnzwcrwcqflnuN7T45R
B3GALT6-B4GALT1^{G}	This study	https://benchling.com/taiki/f/lib_iSYveEoi-chimera_36-41/seq_soOEIt9B-ctsb3galt6-catb4galt1-mng/edit
B4GALT1-B3GALT6^{G}	This study	https://benchling.com/taiki/f/lib_iSYveEoi-chimera_36-41/seq_sfY0E4WX-ctsb4galt1-catb3galt6-mng/edit
ST3GAL4-ST6GAL1^{S}	This study	https://benchling.com/ncu/f/lib_JEsr3knx-swap-mscarlet-st6gal1-st3gals/seq_b5w8PPeg-pcaggs-cts-st34-cat-st61-mscarlet/edit
ST6GAL1-ST3GAL4^{S}	This study	https://benchling.com/ncu/f/lib_JEsr3knx-swap-mscarlet-st6gal1-st3gals/seq_cHM685a0-pcaggs-st3gal4-mscarlet-change-to-st6gal1/edit

Supplementary Table 2. The pairwise distances based on normalized Pearson's correlation coefficient values for MDS analysis

GT	B4GALT1	ST3GAL3	ST3GAL4	ST6GAL1	FUT9	B3GALT6
**B4GALT1**	0	0.25	0.12	0.15	0.02	0.45
**ST3GAL3**	0.25	0	0.185	0.14	0.195	0.26
**ST3GAL4**	0.12	0.185	0	0.09	0.21	0.54
**ST6GAL1**	0.15	0.14	0.09	0	0.14	0.32
**FUT9**	0.02	0.195	0.21	0.14	0	0.63
**B3GALT6**	0.45	0.26	0.54	0.32	0.63	0

The pairwise distances were estimated based on normalized Pearson's correlation coefficient values (r) using the formula: $d = 1 - r$.
